# Supplementary material for: Effects of Endosymbionts on the Nutritional Physiology and Biological Characteristics of Whitefly Bemisia tabaci
Source: Insects. 2025 Jul 9;16(7):703. doi: 10.3390/insects16070703 (PMC12295618; doi:10.3390/insects16070703)
Supplement: Supplementary file 1 [file insects-16-00703-s001.zip › insects-3612273-supplementary.pdf]

Table S1 Two-way analysis of variance on the effects of different host plants on the body length of female and male *B. tabaci* MEAM1

Source	*Df*	MS	F	p
Host plant	5	12540.3	45.7	＜0.001
Gender (female/male)	1	98720.5	359.2	＜0.001
Host plant×Gender	5	630.8	2.3	＜0.05
